# The G0/G1 Switch Gene 2 Is an Important Regulator of Hepatic Triglyceride Metabolism

**DOI:** 10.1371/journal.pone.0072315

**Published:** 2013-08-12

**Authors:** Yinfang Wang, Yahui Zhang, Hang Qian, Juan Lu, Zhifeng Zhang, Xinwen Min, Mingjian Lang, Handong Yang, Nanping Wang, Peng Zhang

**Affiliations:** 1 Cardiovascular Research Center, Hubei University of Medicine, Hubei, China; 2 Department of Physiology, Hubei University of Medicine, Hubei, China; 3 Department of Pathophysiology, Hubei University of Medicine, Hubei, China; 4 Cardiovascular Research Center, Xi’an Jiaotong University School of Medicine, Xi’an, China; University College Dublin, Ireland

## Abstract

Nonalcoholic fatty liver disease is associated with obesity and insulin resistance. Factors that regulate the disposal of hepatic triglycerides contribute to the development of hepatic steatosis. G_0_/G_1_ switch gene 2 (G0S2) is a target of peroxisome proliferator-activated receptors and plays an important role in regulating lipolysis in adipocytes. Therefore, we investigated whether G0S2 plays a role in hepatic lipid metabolism. Adenovirus-mediated expression of G0S2 (Ad-G0S2) potently induced fatty liver in mice. The liver mass of Ad-G0S2-infected mice was markedly increased with excess triglyceride content compared to the control mice. G0S2 did not change cellular cholesterol levels in hepatocytes. G0S2 was found to be co-localized with adipose triglyceride lipase at the surface of lipid droplets. Hepatic G0S2 overexpression resulted in an increase in plasma Low-density lipoprotein (LDL)/Very-Low-density (VLDL) lipoprotein cholesterol level. Plasma High-density lipoprotein (HDL) cholesterol and ketone body levels were slightly decreased in Ad-G0S2 injected mice. G0S2 also increased the accumulation of neutral lipids in cultured HepG2 and L02 cells. However, G0S2 overexpression in the liver significantly improved glucose tolerance in mice. Livers expressing G0S2 exhibited increased 6-(N-(7-nitrobenz-2-oxa-1-3-diazol-4-yl) amino)-6-deoxyglucose uptake compared with livers transfected with control adenovirus. Taken together, our results provide evidence supporting an important role for G0S2 as a regulator of triglyceride content in the liver and suggest that G0S2 may be a molecular target for the treatment of insulin resistance and other obesity-related metabolic disorders.

## Introduction

Hepatic steatosis, also called fatty liver, is caused by the abnormal retention of triglycerides and other fats within liver cells. Obesity, diabetes, and excessive alcohol consumption may contribute to hepatic steatosis [1–3]; more than 50% of all cases with type 2 diabetes have hepatic steatosis. High hepatic triglyceride concentrations are significantly associated with increased fasting insulin levels. It is recognized that hepatic steatosis is a predictor of insulin resistance [1,2]; moreover, hepatic steatosis is also independently associated with the presence and extent of coronary artery disease and other illnesses [4–6].

Within the liver, triglycerides accumulate in lipid droplets, and their deposition is dependent on their biosynthesis and elimination rates [7]. A decreased rate of triglyceride turnover facilitates triglyceride accumulation in the liver [8,9]. A large number of molecules can regulate the generation or disposal of hepatic triglycerides.

G_0_/G_1_ switch gene 2 (G0S2) was initially found to be differentially expressed in lymphocytes during the lectin-induced switch from the G_0_ to G_1_ phase of the cell cycle [10,11]. Both human and mouse G0S2 genes encode a protein of 103 amino acids with 78% sequence homology. Later, it was reported that G0S2 mRNA is also highly expressed in brown and white adipose tissue and up-regulated during growth arrest in 3T3-L1 fibroblasts [12]. The activation of peroxisome proliferator-activated receptors (PPAR)-γ and -α [12], PPAR-β/δ [13], and retinoic acid [14,15] as well as insulin stimulation can upregulate G0S2 expression in 3T3-L1 cells and human acute promyelocytic leukemia cells. Recently, Yang et al. reported that G0S2 inhibits the triglyceride hydrolase activity of adipose triglyceride lipase (ATGL), a major regulator of lipid metabolism in mammals [8], suggesting that G0S2 may play an important role in regulating lipolysis in adipocytes [16]. ATGL is also a major lipase in the liver. However, the expression and function of G0S2 in the liver are still unknown at the present time; therefore, in this study, we examined the biological role of G0S2 in lipid homeostasis in the liver.

## Materials and Methods

### Cell culture and reagents

HepG2, a hepatocytoma cell line, and L02, a normal hepatic cell line (China Center for Type Culture Collection, Wuhan, China), were cultured in Dulbecco’s modified Eagle’s medium (DMEM) supplemented with 10% fetal bovine serum (FBS), 100 U/mL penicillin, 100 µg/mL streptomycin, and 2 mM L-glutamine in 5% CO_2_ at 37° C. DMEM and FBS were purchased from Invitrogen (Carlsbad, CA, USA). Antibodies against G0S2 (N-13), ATGL (F-7), and horseradish peroxidase-conjugated goat anti-rabbit and anti-mouse secondary antibodies were obtained from Santa Cruz Biotechnology (Santa Cruz, CA, USA). FITC-conjugated donkey anti-rabbit and TRITC-conjugated goat anti-mouse antibodies were from Proteintech Group, Inc. (Chicago, IL, USA).

### Adenovirus construction and infection

An adenovirus (Ad) expressing G0S2 was constructed using Adeno-X Expression System 2 following the manufacturer’s instruction (Clontech, CA, USA). Briefly, the full-length coding region of G0S2 was subcloned into the pDNR-CMV vector and transferred to the pLP-Adeno-X-CMV Acceptor Vector via Cre-loxP-mediated recombination. The Ad was amplified and purified using the cesium chloride method. Cells were infected with the Ad at a multiplicity of infection (MOI) of 100.

### Plasmid construction and transfection

We used the pAc-GFP-C1 vector to construct plasmids for the eukaryotic expression of the full-length coding region of the human G0S2 gene. All plasmids were constructed using an In-Fusion™ Dry-Down PCR Cloning Kit according to the manufacturer’s protocol (Clontech, CA, USA). Plasmids were purified using the Promega PureYield Plasmid System. Cultured cells were transfected with the Ac-GFP-G0S2 plasmid using the Lipofectamine™ LTX Reagent (Invitrogen, Carlsbad, CA, USA) and incubated for 24 h. Immunofluorescence was visualized using a laser scanning confocal microscope (Zeiss, Leusden, the Netherlands).

### Animal experiments

All experiments using mice were conducted according to the Guide for the Care and Use of Laboratory Animals published by the US National Institutes of Health (NIH Publications No. 85-23, revised 1996) and approved by the Animal Care and Use Committee of Hubei University of Medicine. Male 4-week-old Swiss mice were used in all experiments. The animals were fed with standard rodent chow and maintained on a 12-h artificial light-dark cycle. Ad-LacZ or Ad-G0S2 (3.0 × 10^9^ pfu) in 100 µL sterilized phosphate-buffered saline (PBS) was delivered by tail vein injection. Diabetes was induced by multiple subdiabetogenic intraperitoneal (i.p.) injections of streptozotocin (STZ) freshly dissolved in 10 mM Na-citrate buffer (pH 4.5) (50 mg STZ/kg body weight daily for 5 consecutive days) (Sigma, MO, USA). The experimental group of mice had food withdrawn for 16 h, with *ad libitum* access to water, previous to sacrifice. Liver tissue was removed for the measurements of organ weight and triglyceride content, RNA and protein extraction, and histological examinations.

### Glucose tolerance test and insulin tolerance test (ITT)

For the oral glucose tolerance test, the mice were fasted overnight, and then, a 2 g/kg body weight glucose solution was administered orally. Blood samples were collected from the tail before and at 30, 60, and 90 min after the glucose challenge for determination of blood glucose levels. For the ITT, the mice were fasted overnight. After measuring body weight and glucose levels, insulin (Sigma) was injected into each mouse i.p. (0.2 U/kg body weight). Blood glucose levels were measured using a glucose meter at 15, 30, 60, and 90 min after the insulin challenge.

### Oil Red O staining

Freshly dissected liver was fixed overnight in 4% paraformaldehyde, cryoprotected in 30% sucrose in PBS for another day, and then frozen in OCT blocks. Cryostat sections 7 µm thick were processed for Oil Red O staining; thereafter, the slices were fractionated with 60% isopropanol for 1 min and washed with PBS for 2 min. To measure the cellular accumulation of neutral lipid droplets, HepG2 and L02 cells were fixed in 4% paraformaldehyde and stained with Oil Red O solution for 30 min at room temperature. The cells were washed with 60% isopropanol. After staining, the cells were washed with PBS to remove unbound dye. To quantify Oil Red O content, the samples were treated with isopropanol and read using a spectrophotometer at 510 nm.

### Hepatic and serum parameters

Triglyceride and cholesterol levels in the liver and cells were measured using colorimetric kits from Abcam (Cambridge, MA, USA) according to the manufacturer’s instructions. Blood for the determination of lipid parameters was obtained from fasted mice. High-density lipoprotein (HDL) cholesterol, low-density lipoprotein (LDL)/very-low-density (VLDL) lipoprotein cholesterol, and ketone bodies in serum samples were evaluated, respectively, using colorimetric kits from BioAssay Systems (Hayward, CA, USA) and a β-Hydroxybutyrate Colorimetric Assay Kit from Abcam (Cambridge, MA, USA) according to the manufacturer’s instruction. A Free Fatty Acid Quantification Kit was purchased from BioVision (Milpitas, CA, USA).

### Co-immunoprecipitation and western blot analyses

For the co-immunoprecipitation assays, 300 µg of total protein extract from mouse livers were immunoprecipitated with the anti-ATGL antibody or pre-immune IgG by using a Dynabeads^®^ Co-Immunoprecipitation Kit (Invitrogen, CA, USA) and then subjected to western blot analysis with the anti-G0S2 antibody. Equal amounts of total protein (30 µg) were also resolved by sodium dodecyl sulfate-polyacrylamide gel electrophoresis (SDS-PAGE) and transferred to nitrocellulose membranes for western blot analysis. The membranes were probed with a primary antibody followed by a secondary antibody conjugated with horseradish peroxidase. The immunocomplexes were visualized with Enhanced Chemiluminescence Plus (Amersham Pharmacia Biotech, NJ, USA).

### Measurement of glucose uptake

Glucose uptake by hepatic cells was assessed *in vivo*. A fluorescent d-glucose analog, 6-(N-(7-nitrobenz-2-oxa-1,3-diaxol-4-yl) amino)-6-deoxyglucose (6-NBDG; Invitrogen, CA, USA), was used to detect glucose uptake by the liver. LacZ- or G0S2-infected mice were fasted overnight and then a glucose solution (2 g/kg) was administered orally. Fifty minutes later, 100 µL of 2 mM 6-NBDG was injected intravenously via the tail vein into each mouse. Another 15 min later, the animals were perfused with PBS via the left ventricle. The liver was minced and homogenized in a lysis buffer supplemented with a protease inhibitor cocktail. The soluble fraction was obtained by centrifugation. The 6-NBDG content in equal amounts of protein lysate was measured by fluorescence intensity (excitation/emission of ~465/540 nm).

### Statistical analysis

Data are expressed as the mean ± standard error of the mean (SEM). Differences were assessed by analysis of the variance or Student’s t-test. *P*-values less than 0.05 were considered statistically significant.

## Results

### Overexpression of G0S2 induced hepatic steatosis

**Figure 1:**
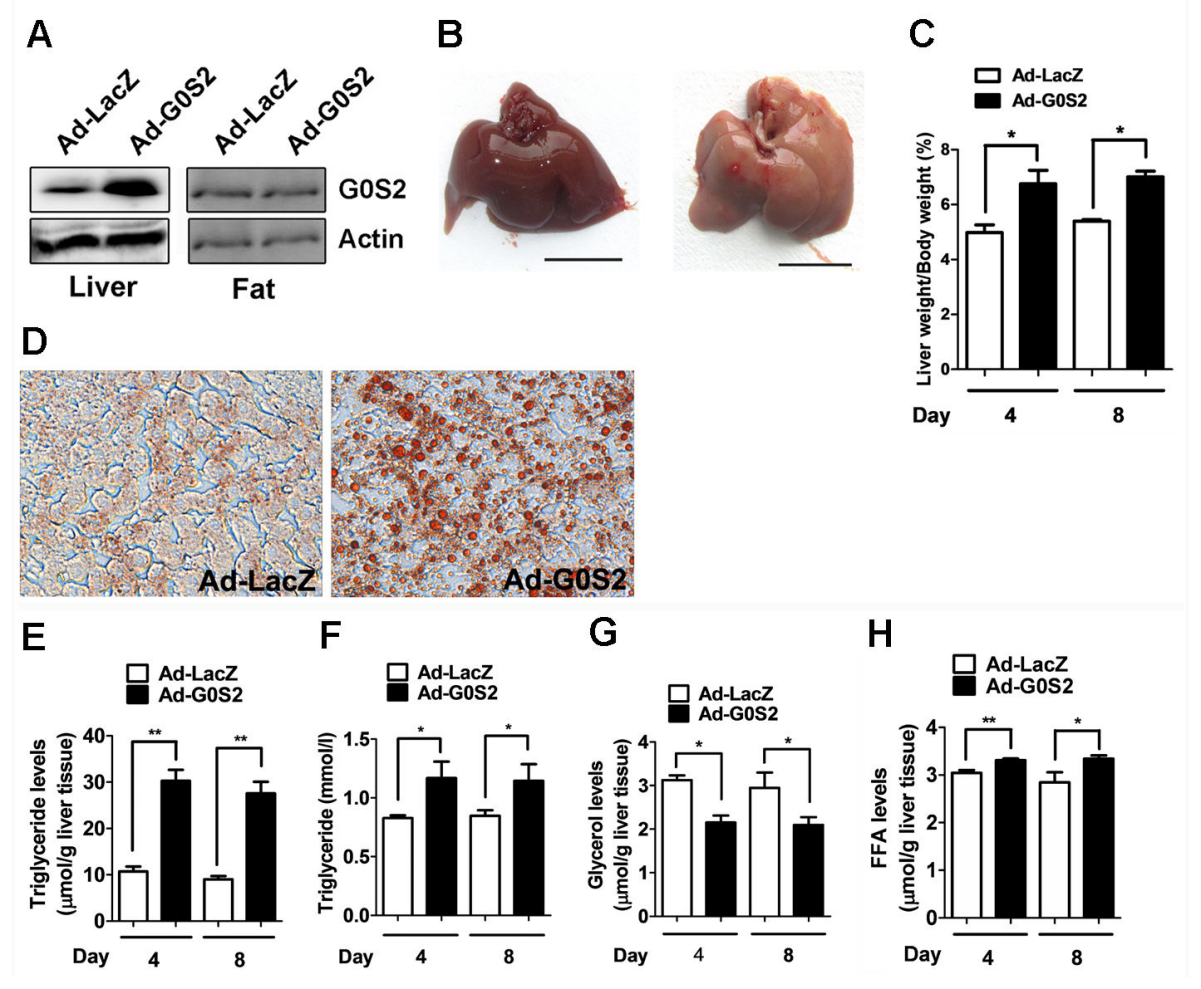
Effects of G0S2 overexpression on lipid accumulation in mice. The mice were injected via the tail vein with an Ad vector at a dose of 3.0 × 10^9^ pfu and were killed at various time points. (A) G0S2 protein levels in the liver and fat tissues were determined with western blotting. (B) Livers of mice infected with Ad-G0S2 (right) or Ad-LacZ (left) for 4 days. Scale bar = 10 mm. (C) The liver weight of mice infected with Ad-LacZ or Ad-G0S2. (D) The liver sections were stained with Oil Red O (magnification 400×). Fasting hepatic and plasma lipid levels were measured. (E) Liver triglyceride content. (F) Plasma triglyceride levels. (G) Liver glycerol content. (H) Liver free fatty acid levels. Each bar represents the mean ± SEM (n = 6). *P < 0.05; **P < 0.01.

To determine the *in vivo* hepatic function of G0S2, a recombinant Ad expressing G0S2 was generated. The mice were injected via the tail vein with the Ad vectors. The expression of G0S2 protein in the liver and fat tissues were verified by western blot analysis (Figure 1A). After 4 or 8 days of infection, the livers were harvested and weighed. The Ad-G0S2-infected mice exhibited typically larger livers than the Ad-LacZ-infected animals (Figure 1B). Liver weight in the Ad-G0S2-infected mice was approximately 1.4-fold greater than that of the Ad-LacZ control mice (Figure 1C). The livers were subsequently sectioned and stained with Oil Red O, which stains neutral lipids. The number and size of lipid droplets were significantly increased in the Ad-G0S2-infected livers compared to the Ad-LacZ-infected livers (Figure 1D).

We further analyzed the effect of G0S2 overexpression on lipid content. The triglyceride content of Ad-G0S2-infected livers was significantly increased compared to the Ad-LacZ-infected livers (30.30 ± 4.72 µmol/g tissue vs. 10.71 ± 2.14 µmol/g tissue at day 4; 27.57 ± 5.05 µmol/g tissue vs. 9.00 ± 1.40 µmol/g tissue at day 8, respectively) (Figure 1E). The Ad-G0S2-infected animals also exhibited elevated plasma triglyceride levels (Figure 1F). Glycerol levels in the Ad-G0S2-infected livers were lower than in the control livers (Figure 1G); however, the Ad-G0S2-infected animals exhibited elevated hepatic free fatty acid levels (Figure 1H). The levels of free cholesterol and esterified cholesterol in the liver remained unchanged (data not shown). These results suggest that G0S2 overexpression induced the accumulation of triglycerides and promoted fatty liver formation.

### Effect of G0S2 on the levels of plasma HDL cholesterol, LDL/VLDL cholesterol, and ketone bodies

**Figure 2:**
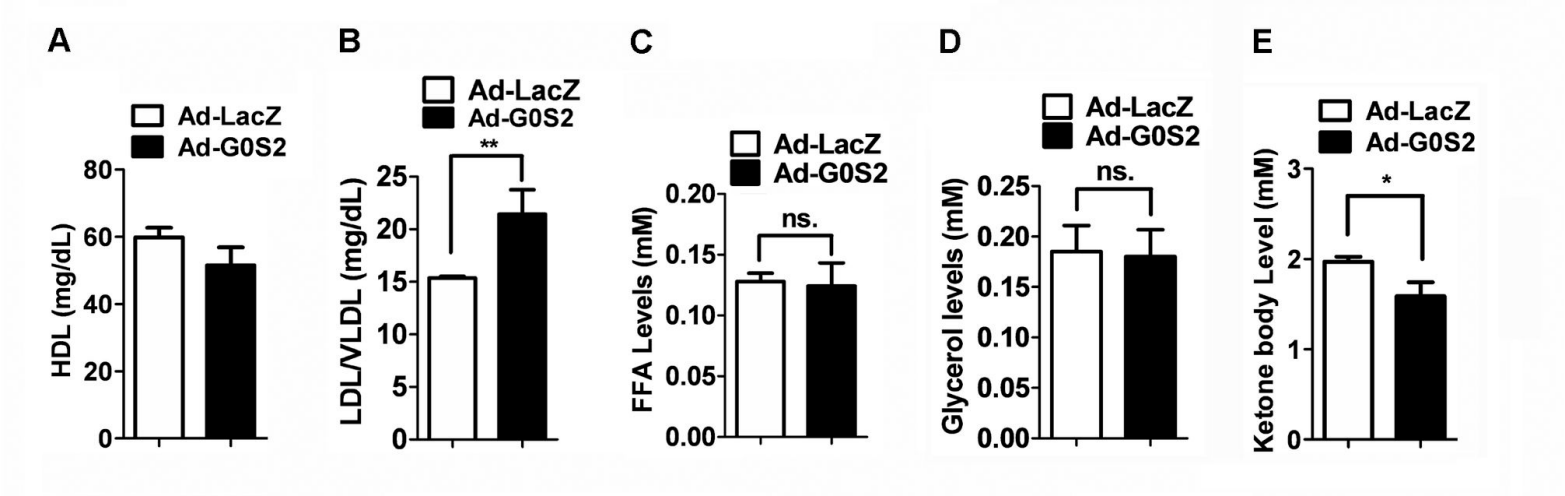
Plasma HDL cholesterol, LDL/VLDL cholesterol, and ketone body levels in Ad-infected mice. Mice were infected with the indicated adenovirus vector for 4 days. Fasting plasma HDL cholesterol (A), LDL/VLDL cholesterol (B), free fatty acids (C), glycerol (D) and ketone body (E) levels were detected. Each bar represents the mean ± SEM (n = 6). *P < 0.05; **P < 0.01; ns, not significant.

Systemic free fatty acids are the major substrate for VLDL-TAG and ketone body 3-hydroxybutyrate production. HDL cholesterol and LDL cholesterol are two major components of cholesterol. We first examined the effect of G0S2 overexpression on HDL cholesterol and LDL/VLDL cholesterol levels in plasma. The resulting data showed that G0S2 overexpression increased LDL/VLDL cholesterol levels, while it slightly decreased HDL cholesterol levels in plasma (Figure 2A, 2B). However, the levels of plasma free fatty acids and glycerol remained unchanged (Figure 2C, 2D). Reportedly, ATGL regulates fatty acid oxidation. We further investigated the effect of G0S2 overexpression on ketone body production. Reduced ketone body production was observed in the mice injected with Ad-G0S2 compared to the control mice (Figure 2E).

### G0S2 was coupled with ATGL in the mouse liver

**Figure 3:**
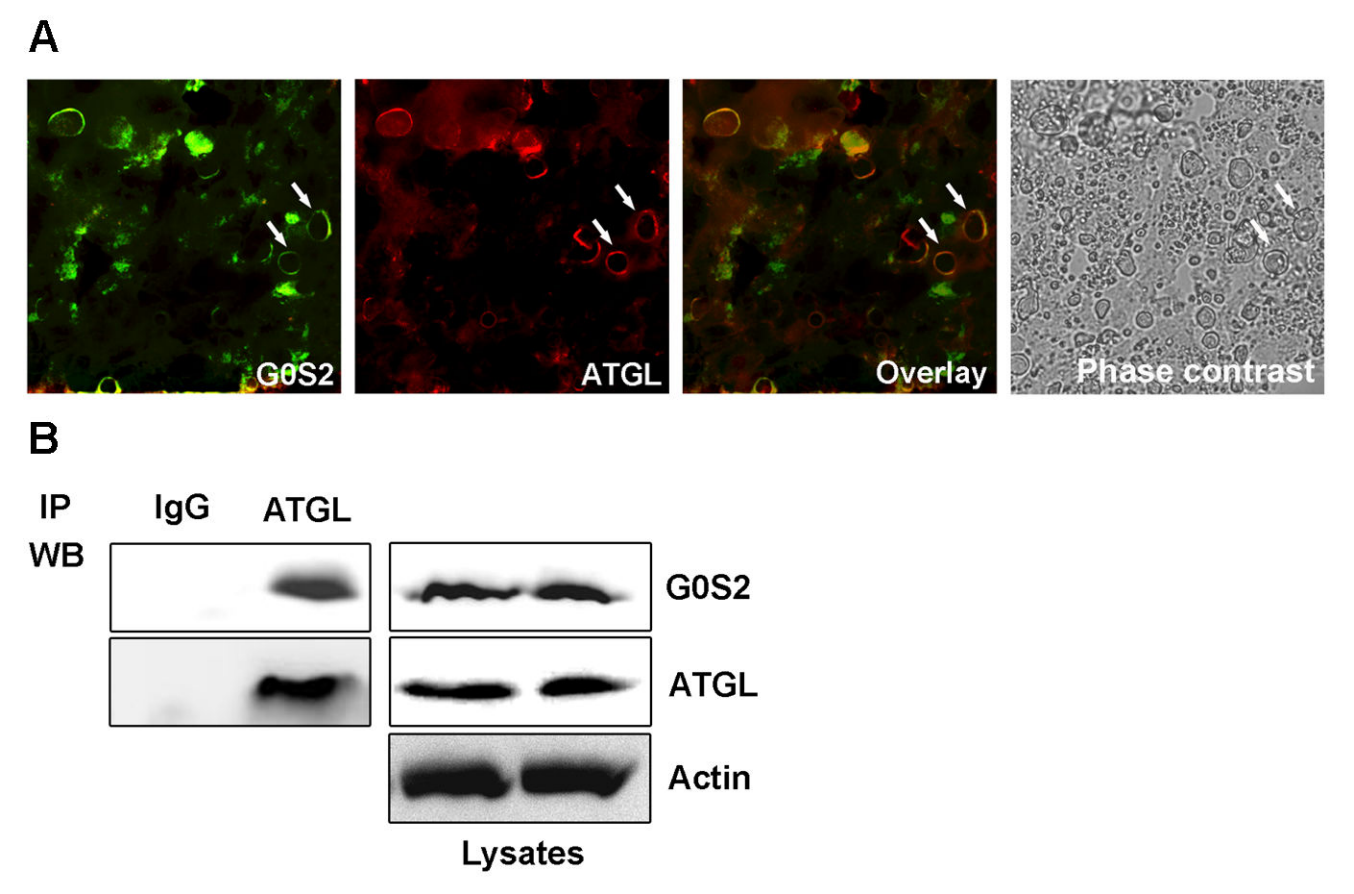
G0S2 binds to ATGL in the liver. Mice were injected via the tail vein with an Ad-G0S2 vector and killed 4 days later. (A) Immunofluorescent analysis of liver sections with the anti-G0S2 and anti-ATGL antibodies. Bound primary antibodies were visualized, respectively, with FITC-conjugated anti-rabbit IgG or TRITC-conjugated anti-mouse IgG. The sections were visualized under laser confocal microscopy. LDs in the livers were visualized under a phase-contrast microscope. The arrows indicate positive staining. Scale bar = 10 µm. (B) Anti-G0S2 and anti-ATGL western blot (WB) analyses were performed on ATGL or mouse monoclonal IgG immunoprecipitates (IP) prepared with the anti-ATGL antibody or IgG affinity Dynabeads (left panel). To control for equal loading, equal amounts by volume of crude extract for the immunoprecipitation experiments were loaded onto the SDS-PAGE gel for immunoblotting (right panel).

Since G0S2 functionally associates with ATGL and regulates its action in adipocytes, we examined whether G0S2 physically interacts with ATGL in the mouse liver. The subcellular localization of G0S2 and ATGL was determined by using immunofluorescence staining. LDs in the liver were visualized under a phase-contrast microscope and confirmed using Oil Red O staining. G0S2 was found to be localized at the surface of lipid droplets and co-localized with ATGL (Figure 3A). To confirm this observation, we evaluated the interaction between G0S2 and ATGL using a co-immunoprecipitation assay. Immunoblotting of the ATGL-immunoprecipitates from the liver lysates showed that G0S2 co-precipitated with ATGL (Figure 3B), confirming that G0S2 interacts with ATGL in the liver.

### Changes in the expression levels of mRNAs encoded by PPAR target genes

**Figure 4:**
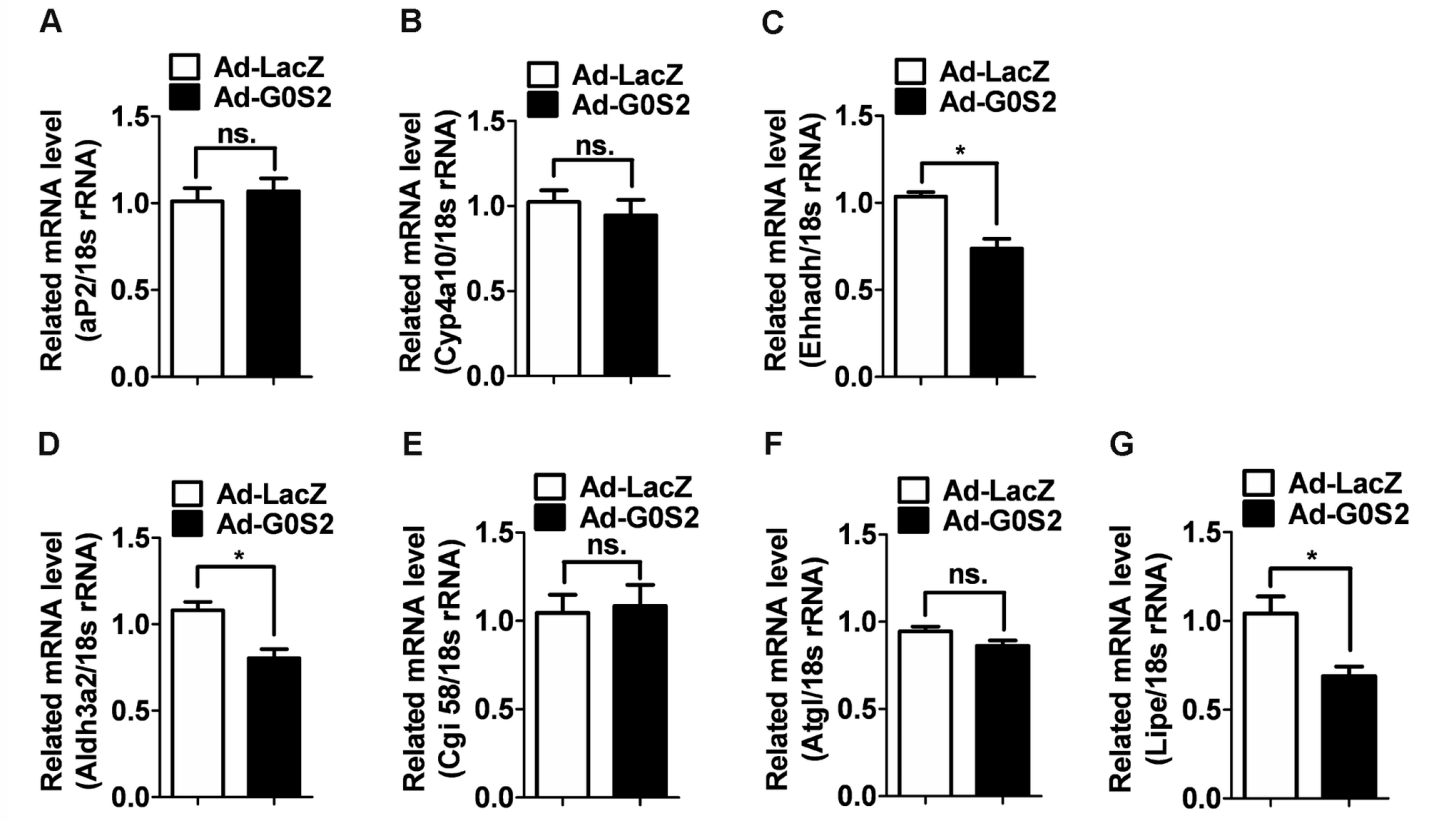
Changes in PPAR-α activated gene expression in mice overexpressing G0S2 in the liver. Mice were injected via the tail vein with the Ad-G0S2 vector and killed 4 days later. Relative mRNA levels of aP2 (A), Cyp4a10 (B), Ehhadh (C), Aldh3a2 (D), Cgi58 (E), Atgl (F), and Lipe (G) in the liver after fasting. *P < 0.05, compared with the Ad-LacZ-infected mice (n = 5); ns, not significant.

Lipolysis of cellular triglycerides by ATGL generates mediators involved in the generation of lipid ligands for PPAR activation. ATGL deficiency in mice can decrease the mRNA levels of PPAR-α and PPAR-δ target genes in cardiomyocytes. We examined the expression of aP2, Cyp4a10, Ehhadh, Fgf21, and Aldh3a2 mRNA in the liver. The resulting data showed that the mRNA levels of Ehhadh and Aldh3a2 in the liver were reduced in the Ad-G0S2-infected mice compared to the control mice. G0S2 overexpression did not alter aP2 or Cyp4a10 expression (Figure 4A–D). We further investigated the effect of G0S2 overexpression on the mRNA levels of genes involved in lipolysis, e.g., Lipe and Cgi58. The data showed that hepatic G0S2 overexpression in mice decreased the mRNA levels of Lipe, but did not alter Cgi58 or Atgl expression (Figure 4E–G).

### G0S2 overexpression improves glucose tolerance

**Figure 5:**
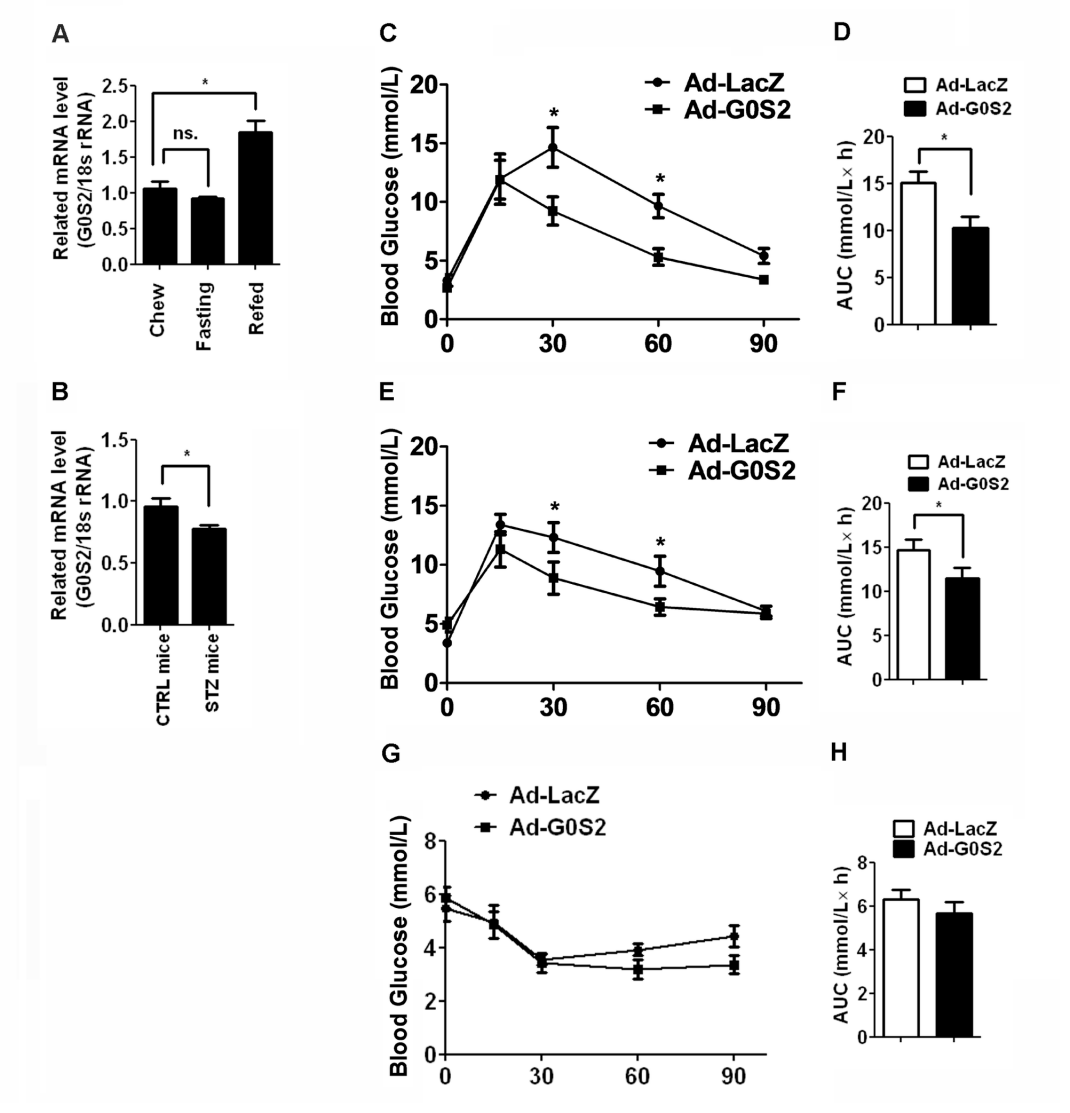
Overexpression of G0S2 improves glucose tolerance in mice. (A) G0S2 mRNA levels in liver following *ad libitum* feeding, a 12 h fast, or *ad libitum* refeeding for 12 h following the 12 h fast. (B) The expression of G0S2 mRNA in the liver of control mice and mice treated with STZ. Blood glucose levels and the inverse integrated area under the glucose curve of mice at 4 (C, D) or 8 (E, F) days after injection with Ad-LacZ or Ad-G0S2. Change in plasma glucose levels (G) and the mean area under the curve (H) after an ITT in mice. *P < 0.05, compared with the Ad-LacZ-infected mice (n = 6).

Hepatic steatosis is associated with insulin resistance. To study the regulation of G0S2 expression by insulin, we performed a fasting/refeeding experiment. G0S2 mRNA levels fell slightly upon fasting and were restored by refeeding (Figure 5A). Furthermore, STZ injection caused G0S2 levels to decrease by 30% in the liver (Figure 5B). We next investigated the ability of G0S2 overexpression to influence glucose tolerance *in vivo*. We found that overexpression of G0S2 in the liver significantly improved glucose tolerance (*P* < 0.05 at 30 and 60 min) at days 4 (Figure 5C, 5D) and 8 (Figure 5E, 5F) post-infection. Fasting glucose levels were not significantly different between the Ad-G0S2- and Ad-LacZ-infected groups. These results demonstrate that G0S2 modulates glucose as well as lipid homeostasis *in vivo*. We further carried out an ITT in mice fed a chow diet. Overexpression of G0S2 in the liver resulted in a slightly, but not significantly, hypoglycemic response to insulin compared with the control mice (Figure 5G, 5H). Therefore, hepatic G0S2 overexpression improves glucose disposal *in vivo*.

### G0S2 overexpression increases hepatic glucose uptake

**Figure 6:**
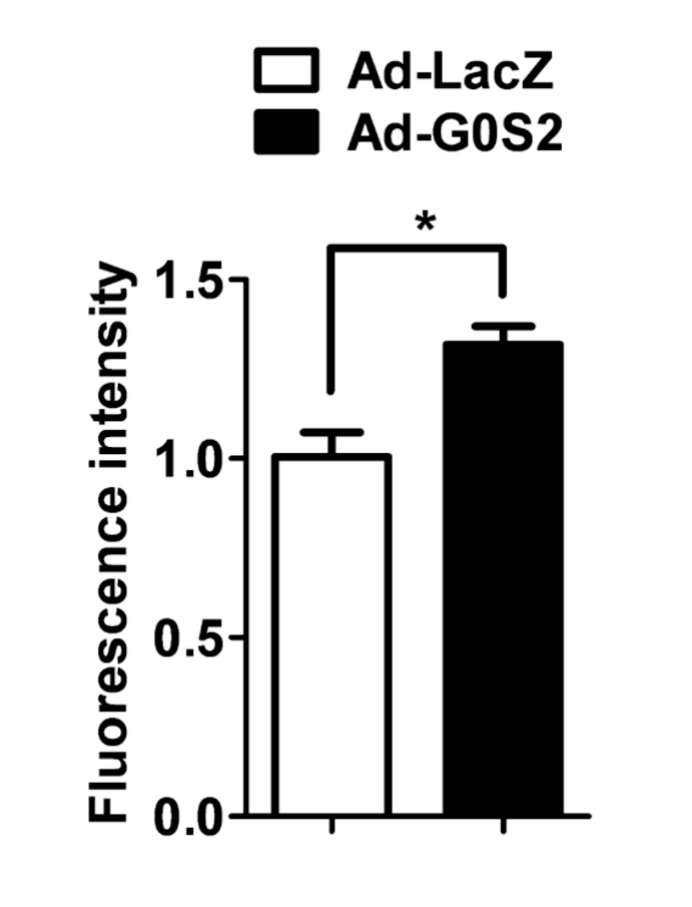
Effects of G0S2 overexpression on hepatic glucose uptake. The fluorescence intensity of 6-NBDG in Ad-G0S2- or Ad-LacZ-infected livers was detected. Each bar represents the mean ± SEM (n = 5). *P < 0.05.

To further assess whether G0S2 affect glucose uptake by the liver, we examined the fluorescence intensity of 6-NBDG in equal amounts of hepatic protein lysate. It was shown that the fluorescence intensity of 6-NBDG was significantly increased in the Ad-G0S2-infected livers compared to the Ad-LacZ-infected livers (Figure 6). These data indicate that G0S2 enhances glucose tolerance by increasing hepatic glucose uptake.

### G0S2 resulted in lipid accumulation in hepatocytes *in vitro*

**Figure 7:**
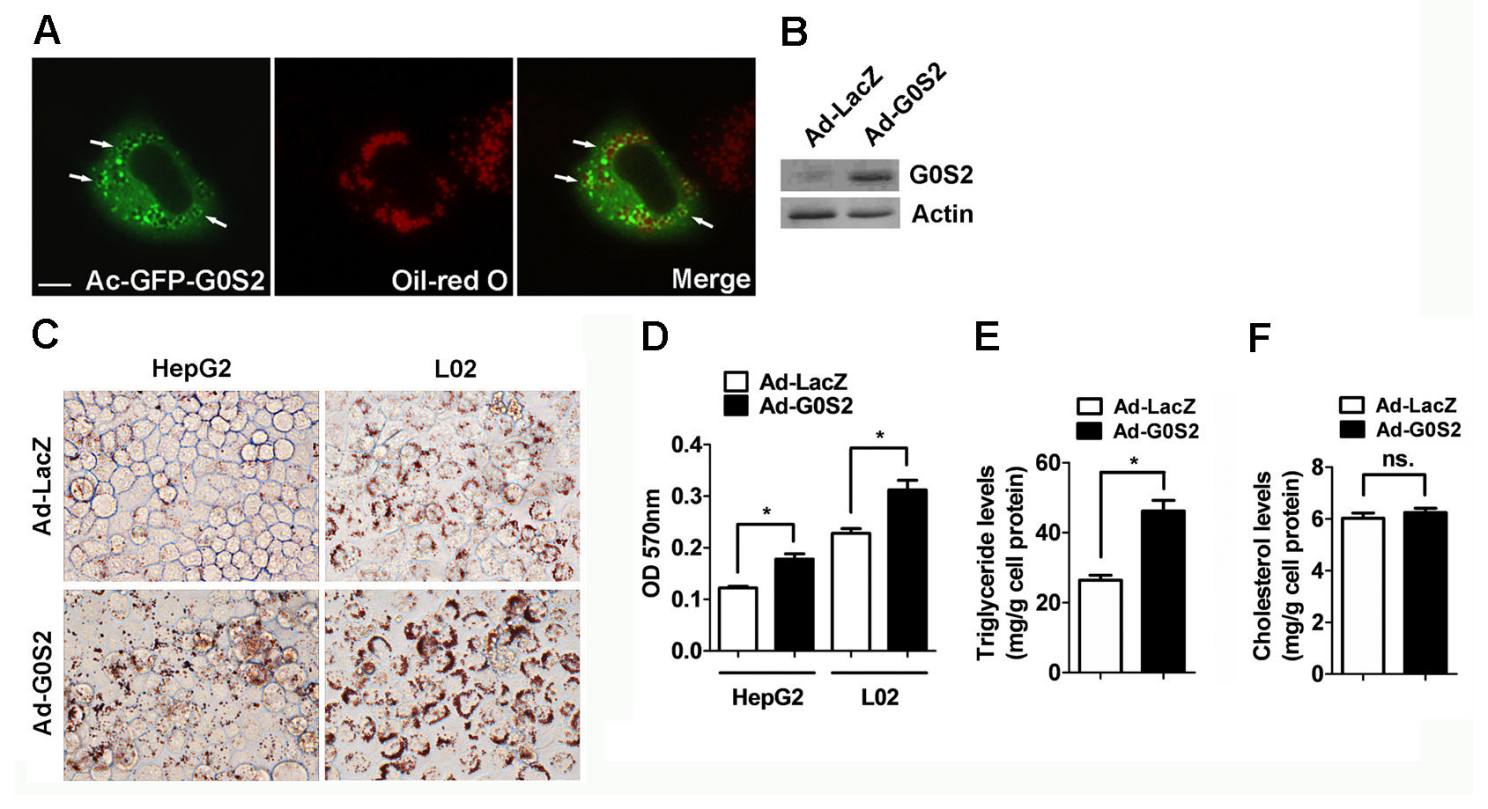
G0S2 causes lipid accumulation in hepatocyte cell lines. (A) L02 cells were transfected with the Ad-GFP-G0S2 plasmid and incubated under normal growth conditions for 24 h. The cells were fixed and stained with Oil Red O and then observed under fluorescence microscopy. (B) G0S2 protein levels in the L02 cells were determined with western blotting. (C) HepG2 or L02 cells were infected with Ad-LacZ or Ad-G0S2 for 48 h before they were fixed and stained with Oil Red O. Representative images are shown (magnification 400×). (D) Oil Red O content in HepG2 and L02 cells. (E and F) Triglyceride and cholesterol levels in L02 cells. Each bar represents the mean ± SEM (n = 3). *P < 0.05; ns, not significant.

Whether G0S2 directly modulates the lipid content of hepatocytes was further examined *in vitro*. The GFP-G0S2 fusion protein was expressed in L02 cells; lipid droplets were stained with Oil Red O. Here, we found that GFP-G0S2 protein was located in the proximity of the lipid droplets of L02 cells (Figure 7A). The overexpression of G0S2 protein in the Ad-infected L02 cells was verified by western blot analysis (Figure 7B). In accordance with the *in vivo* results, G0S2 overexpression significantly increased neutral lipid content in L02 and HepG2 cells compared with control cells (Figure 7C, 7D). Cholesterol and triglyceride content in the L02 cells was analyzed subsequently. The resulting data showed that G0S2 overexpression caused an approximately 1.5-fold increase in cellular triglyceride content, but did not change cellular cholesterol levels in hepatocytes (Figure 7E, 7F).

## Discussion

In the present study, we found that the overexpression of G0S2 induces triglyceride accumulation in the mouse liver and results in the development of hepatic steatosis. G0S2 overexpression can increase LDL/VLDL cholesterol levels and decrease HDL cholesterol and ketone body levels in the blood. G0S2 couples with ATGL and promotes lipid accumulation in hepatocytes. However, G0S2 overexpression in the liver has beneficial effects on glucose homeostasis. These findings suggest that G0S2 is a crucial regulator of hepatic triglyceride metabolism.

Although G0S2 was first identified as a potential cell cycle regulator, it has been shown that G0S2 expression is restricted to forming brown adipose tissue and liver during murine embryonic development [17]. Similarly, Zandbergen et al. showed that G0S2 is a PPAR target gene; G0S2 mRNA levels were the highest in brown and white adipose tissue and were greatly up-regulated during the adipogenesis of mouse 3T3-L1 cells [12]. These data give a clue that G0S2 may participate in lipid metabolism. Yang et al. recently reported that G0S2 localizes to lipid droplets and prevents their ATGL-mediated turnover in HeLa cells and adipocytes [16]. The liver and adipose tissue are the main sites of triglyceride synthesis. In the present study, we provided direct evidence that G0S2 induced liver triglyceride accumulation and severe hepatic steatosis under basal conditions.

It is known that PPAR agonists decrease hepatic lipid content in humans and rodents [18,19]. A therapeutic dose of a PPAR-α agonist increased the liver triglyceride levels in mice [20]. Fenofibrate treatment of rats resulted in a 50% increase in liver triglyceride levels [21]. Moreover, bezafibrate, an agonist of murine PPAR-α and a partial agonist of PPAR-γ, increased cellular triglyceride mass in primary rat hepatocytes [22]. On the contrary, a PPAR-α agonist increased hepatic triglyceride content less in animals that received a high-fat diet [23–26]. In this study, we also found that the mRNA expression of G0S2 in the liver was up-regulated (approximately 2-fold induction) by fenofibrate (data not show). Therefore, G0S2 is likely to play a role in the maintenance of the liver triglyceride pool under physiological conditions. It is possible that the modulation of liver triglyceride levels by PPAR-α agonists is at least partially achieved via the induction of G0S2. Moreover, it is likely that G0S2 is also a negative regulator of PPAR agonists and may counterbalance the triglyceride lowering effects of PPAR under pathophysiological conditions.

Fatty acids are broken down enzymatically by β-oxidation to form acetyl-CoA. The ATGL-mediated hydrolysis of triglycerides generates fatty acids and diacylglycerol (DAG). Reportedly, fatty acid oxidation was increased by ATGL overexpression and decreased by ATGL knockdown [27]. The current study provided evidence that the overexpression of G0S2 in the liver partly decreased the levels of serum ketone bodies. Given that G0S2 interacts directly with ATGL to suppress its enzymatic activity, it is speculated that G0S2 mediated the down-regulation of ketone body production by suppressing free fatty acid levels or hepatic fatty acid oxidation. Reportedly, the lipolysis of cellular triglycerides by ATGL generates mediators that are involved in the generation of lipid ligands for PPAR activation. ATGL could also influence PPAR-α activity independent of ligand-induced activation [28]. We found that the administration of Ad-G0S2 decreased Ehhadh and Aldh3a2 mRNA levels, but did not significantly change Acot1 or Cyp4a10 mRNA expression in the liver. Therefore, it is suggested that G0S2 influenced ketone body formation partly independently of PPAR ligand-induced activation.

Endogenous ATGL is localized to the external surface of lipid droplets in adipocytes, and it plays a key role in lipid droplet/adiposome degradation in mammalian cells. G0S2 couples with ATGL and prevents ATGL-mediated basal lipid droplet degradation in adipocytes and HeLa cells [16]. Recent work by Lu et al. demonstrated that G0S2 binds to ATGL in a manner that is dependent on the intact 3-dimensional structure of the patatin-like domain and may block the substrate accessibility of ATGL [29]. In this study, we demonstrated that G0S2 and ATGL were colocalized on the surface of lipid droplets. Co-immunoprecipitation confirmed that G0S2 binds directly with ATGL in hepatocytes. A previous study showed that hepatic overexpression of ATGL stimulates the direct release of free fatty acids. Here, we found that Ad-G0S2-infected animals exhibit elevated hepatic free fatty acid levels. We hypothesize that increased hepatic triglyceride storage pools and decreased direct release of free fatty acids could result in elevated hepatic free fatty acid levels. Furthermore, glycerol levels in Ad-G0S2-infected livers were decreased compared with controls. Collectively, these observations indicate that G0S2 can decrease triglyceride degradation in hepatocytes through binding with ATGL and inhibiting its activity.

It is worth noting that G0S2-induced hepatic steatosis was very potent (by 3-fold) *in vivo*, but to a lesser extent (1.5-fold) in hepatocytes *in vitro*. Lipid droplets of Ad-G0S2-infected livers *in vivo* were also significantly larger than those seen in Ad-G0S2-infected HepG2 and L02 cells *in vitro*. To address this discrepancy, physiological concentrations of oleic acid were added to the culture media; however, G0S2-induced hepatic lipid accumulation in hepatocytes *in vitro* was still much weaker than *in vivo* (data not shown). Furthermore, a high MOI still could not increase lipid accumulation in hepatocytes. The differences between the *in vitro* and *in vivo* results may indicate that internal conditions can modulate G0S2 activity and the global metabolic effects of G0S2 may also contribute to the development of hepatic steatosis.

A number of studies have documented a strong relationship between hepatic steatosis and insulin resistance. Hepatic steatosis is thought to be part of metabolic syndrome [2,30]. G0S2 expression in fat tissue was found to be decreased in db/db mice and mice fed with a high-fat diet. We reported here that STZ treatment also decreased the expression of G0S2 mRNA in the liver. Insulin increased but TNF-α decreased the G0S2 levels in 3T3-L1 adipocytes [16]. On the contrary, G0S2 was also identified as a target of carbohydrate response element-binding protein/Max-like factor X [31]. Its mRNA expression in hepatocytes was upregulated by high glucose. Here, we unexpectedly found that the overexpression of G0S2 in the liver increased glucose disposal *in vivo*. Discrepant effects on insulin sensitivity and hepatic steatosis have been observed in several recent studies. Reportedly, liver-specific Akt activation [32], angiopoietin-like protein 4 overexpression [33], and inhibition of phosphatase and tensin homolog [34] improved glucose tolerance, but induced hyperlipidemia and hepatic steatosis in mice. Moreover, G0S2 attenuates ATGL action. ATGL null mice store vast amounts of triacylglycerol in key glucoregulatory tissues, yet exhibit enhanced insulin sensitivity and glucose tolerance [35]. Previous evidence also showed that the reduced availability of ATGL-derived free fatty acids lead to increased glucose use [36]. Furthermore, ATGL cleaves the first fatty acid to create DAG. Reportedly, ATGL overexpression could promote DAG accumulation and disrupt insulin signaling and action in myotubes [37]. Hepatic DAG content was found to be strongly correlated with the activation of hepatic PKCε, which impairs insulin signaling [38]. Recently, Ong et al. reported that hepatic ATGL knockdown decreased glucose production and increased glucose oxidation in the liver [28]. Thus, G0S2 could probably improve glucose tolerance by inhibiting ATGL activity. However, it seems that hepatic glucose uptake is minimal compared to peripheral glucose uptake. Furthermore, we also have no idea whether G0S2 overexpression regulates hepatic insulin signaling activation. Therefore, these data indicate that hepatic glucose uptake at least partially contributed to the enhanced glucose tolerance of mice infected with Ad-G0S2. The detailed mechanisms for the improvement of glucose homeostasis by G0S2 still remain to be elucidated.

In summary, our present study provided experimental evidence supporting an important role for G0S2 as a regulator of triglyceride content in the liver. Further investigations are needed to address the functions of G0S2 in the pathophysiology of obesity-related hepatic steatosis in humans.
